# Phenothiazines to Treat Alzheimer’s Disease

**DOI:** 10.1093/geroni/igab046.2417

**Published:** 2021-12-17

**Authors:** Rachel Litke, Bik Tzu Huang, Damian Gonzalez, Martine Rampanana, Nicholas Grimaldi, Charles Mobbs

**Affiliations:** 1 icahn school of medicine at mount sinai, New York, New York, United States; 2 Icahn School of Medicine at Mount Sinai, New York, New York, United States; 3 Icahn School of Medicine at Mount Sinai, new york, New York, United States

## Abstract

Current treatments of Alzheimer’s Disease (AD) are largely ineffective and do not address underlying pathophysiological processes. The model organism C. elegans has been successfully used to discover compounds to treat human diseases, some now in clinical trials. To develop novel drugs and explore pathways to treat AD, we took on a forward pharmacological approach with a C. elegans model for AD, completed with studies to expand results to lifespan as well as healthspan. We screened 2560 drugs from the Microsource Spectrum library for their ability to delay proteotoxicity (indicated by paralysis) in an Abeta transgenic C. elegans muscle model of AD (CL2006) in liquid medium. Among the most protective drugs were phenothiazines, which are orally active and cross the blood-brain barrier, desirable properties of drugs to treat AD. 80 phenothiazines congeners were further assessed; 60% were protective in CL2006 worms. 9/20 tested phenothiazines increased lifespan in N2 worms and 2/3 phenothiazines tested promoted significantly higher pharyngeal pumping rates compared with control till day 10 of adulthood in N2 worms. 2 of the drugs were protective in the C. elegans neuronal model of AD. This phenotypic screening approach led to the discovery of potential drugs to treat AD. These phenothiazines protect against Abeta toxicity, and assessment of efficacy to protect against other forms of proteotoxicity are ongoing. These studies suggest the utility of C. elegans to discover drugs to treat human diseases. Future studies will assess molecular mechanisms mediating the protective effects of these compounds.

